# Faculty Perspectives on Integrating Artificial Intelligence Into Orthodontic and Interdisciplinary Dental Education: Opportunities, Challenges, and Strategies

**DOI:** 10.7759/cureus.92877

**Published:** 2025-09-21

**Authors:** Joyshree Chutia, Rupali Rupali, Megha Jain, Angel Angel, Priyanka Agarwal, Chand Sawhney

**Affiliations:** 1 Department of Dentistry, Tezpur Medical College and Hospital, Tezpur, IND; 2 Department of Orthodontics, Caps ‘N’ Crown Dental Care, Kharar, IND; 3 Department of Dentistry, Shri Balaji Action Medical Institute, New Delhi, IND; 4 Department of Pedodontics and Preventive Dentistry, Desh Bhaghat Dental College and Hospital, Mandi Gobindgarh, IND; 5 Department of Orthodontics, Mahatma Gandhi Dental College and Hospital, Jaipur, IND; 6 Department of Orthodontics, Maharana Pratap College of Dentistry and Research Centre, Gwalior, IND

**Keywords:** artificial intelligence, curriculum, interdisciplinary, orthodontics, perception, survey

## Abstract

Introduction: Artificial intelligence (AI) is transforming dental education by enhancing diagnostic precision and personalized learning. This study aimed to investigate faculty perspectives on integrating AI into orthodontic and interdisciplinary dental curricula and identifying opportunities, challenges, and strategies to prepare students for technology-driven practice. The objectives included exploring AI’s potential in enhancing diagnostic tools and learning systems, identifying barriers such as faculty training gaps and ethical concerns, and developing evidence-based strategies for AI adoption.

Materials and methods: A cross-sectional survey was conducted in the Department of Orthodontics. A structured questionnaire developed by a multidisciplinary team comprised 12 items across three sections: opportunities, challenges, and strategies, rated on a numerical scale (-1 = Disagree, 0 = neutral and +1 = Agree). After pilot testing (Cronbach’s alpha = 0.82), the survey was distributed via WhatsApp to 410 faculty members from North Indian dental colleges, targeting orthodontists and related specialties with at least two years of teaching experience. Data were analyzed using independent t-tests and one-way analysis of variance (ANOVA) for comparison (p < 0.05).

Results: A total of 250 (61%) participants responded to the survey. The study population included 140 (56%) males with a mean age of 38.11 ± 5.08 years and 110 (44%) females, with orthodontists as the largest specialty group. Females reported higher opportunity (mean = 3.14 vs. 2.82, p = 0.048) and challenge scores (mean = 3.41 vs. 3.14, p = 0.029) than males, with no significant difference in strategy perceptions (p = 0.179). Full-time academicians noted greater challenges (p = 0.013) than clinician-academicians. Orthodontists showed the highest opportunity scores (mean = 3.33) and lowest challenge scores (mean = 3.06), whereas prosthodontists reported the highest challenges (mean = 3.57, p = 0.004). Age and experience were positively correlated with opportunity and strategy perceptions, with weaker negative correlations with challenges.

Conclusion: The findings revealed strong faculty support for AI integration, with perspectives influenced by sex, specialty, and role. Tailored training and curriculum redesign are needed to address these barriers and enhance AI adoption in dental education.

## Introduction

The integration of artificial intelligence (AI) into healthcare has ushered in transformative advancements, with dentistry, particularly orthodontics and interdisciplinary dental fields, experiencing significant impacts [1]. AI technologies, including machine learning for cephalometric analysis, predictive modeling for treatment planning, and the automated design of orthodontic appliances, are revolutionizing clinical workflows by enhancing precision and efficiency [2]. As dental education traditionally focuses on manual skills and evidence-based practice, the rise of AI necessitates a paradigm shift in curricula to prepare students for a technology-driven future [3]. Faculty members, as key stakeholders in shaping educational frameworks, are critical to this transition, offering unique perspectives on balancing innovation with foundational training [4].

AI presents numerous opportunities to enhance orthodontic education [1,4]. Tools such as AI-driven virtual simulations enable immersive training in complex procedures, such as bracket positioning and malocclusion diagnosis, fostering collaboration across disciplines such as radiology and prosthodontics [2]. Adaptive learning platforms powered by AI can tailor educational content to individual student needs, thereby accelerating the mastery of data-driven diagnostics and ethical AI applications [5]. Furthermore, AI facilitates the integration of research into curricula, empowering the faculty to guide students in leveraging large datasets for evidence-based orthodontic strategies, thus bridging theoretical knowledge with clinical practice [5].

Despite these opportunities, several challenges persist. Faculties often lack sufficient training in AI tools, leading to hesitancy or ineffective implementation. Ethical concerns, such as data privacy, algorithmic bias, and the risk of over-reliance on technology, threaten to undermine the humanistic aspects of dental care [4,6]. Additionally, integrating AI into interdisciplinary curricula faces obstacles, such as resource disparities and outdated accreditation standards. These challenges highlight the need for strategic faculty development and curriculum redesign to ensure effective adoption of AI. This study explored faculty perspectives to navigate these opportunities and challenges, focusing on orthodontic and interdisciplinary dental education to propose actionable strategies for curriculum enhancement.

The aim of this study was to investigate faculty perspectives on integrating AI into orthodontic and interdisciplinary dental curricula, identifying opportunities for innovation and strategies to address barriers, and ultimately preparing students for technology-driven dental practice. The objectives were to explore opportunities for AI to enhance orthodontic education, to identify challenges in AI integration, and to develop evidence-based educational strategies for incorporating AI, including curriculum redesign, faculty development programs, and interdisciplinary collaboration frameworks.

## Materials and methods

This study employed a cross-sectional survey design, utilizing a structured questionnaire to collect data on the opportunities, challenges, and educational strategies related to AI adoption. The study was conducted in the Department of Orthodontics, Mahatma Gandhi Dental College and Hospital, Jaipur, India, between January 2025 and March 2025. Ethical approval (MGDCH/Dental/2024/SS61) was obtained from the Institutional Ethics Committee (IEC) to ensure compliance with ethical standards, including informed consent, anonymity, and data confidentiality. Informed consent was obtained from all participants through a digital consent form embedded in the Google Forms questionnaire, ensuring voluntary participation, anonymity, and right to withdraw.

The questionnaire was collaboratively developed by a multidisciplinary team of faculty members comprising four orthodontists and one pedodontist from different dental colleges across different regions in India, including Delhi, Madhya Pradesh, Assam, Punjab, and Rajasthan, to ensure diverse perspectives and regional relevance. The team engaged in virtual meetings via Zoom to discuss AI integration challenges and opportunities, sharing insights from their respective specialties and institutional contexts. A shared online platform facilitated iterative drafting and feedback, with each member contributing to item formulation and cultural relevance. Regular email correspondence and a centralized protocol ensured alignment, while a lead investigator coordinated revisions to maintain consistency and address regional variations in dental education infrastructure. The development process began with a comprehensive literature review of AI in dental education, drawing from sources such as systematic reviews on AI's role in orthodontics and educational challenges. The key themes identified included diagnostic enhancements, ethical concerns, and curricular strategies, which informed the questionnaire's content. 

The questionnaire was structured to align with the study's objectives and comprised 12 items organized into three distinct sections. Section 1, "Opportunities for AI integration" (Items 1-4), evaluated faculty perceptions of AI's potential benefits in orthodontic and interdisciplinary dental education, focusing on areas such as improved diagnostic accuracy through AI-driven tools, enhanced practical skills training via simulations, support for personalized learning pathways, and preparation of students for modern, technology-driven dental practice. Section 2, "Challenges of AI integration" (Items 5-8), addressed perceived barriers, including limited faculty training in AI tools, technical infrastructure constraints, ethical concerns such as data privacy, and the risk of overreliance on AI, potentially diminishing students' critical thinking skills. Section 3, "Educational strategies for AI integration" (Items 9-12), explored solutions to facilitate effective AI adoption, such as the implementation of faculty development programs, the incorporation of AI literacy as a core curricular component, collaborative workshops with technology experts, and the use of pilot programs to test AI tools before full integration. Each section was designed to comprehensively capture faculty perspectives on opportunities, challenges, and strategies for embedding AI in dental curricula.

Each item was phrased as a statement and rated on a three-point numerical scale: -1 = disagree, 0 = neutral, and 1 = agree. The minimum score for each category was -4 and maximum was 4. This scale was selected for its simplicity and suitability for attitudinal surveys (directional positive or negative), reducing respondent burden while allowing a clear differentiation of opinions. The questionnaire included introductory instructions emphasizing anonymity and the study's purpose, and was concluded with a thank-you note. The total estimated completion time was 10 min.

Prior to full distribution, the questionnaire was pilot-tested to evaluate its clarity, reliability, and validity. A convenience sample of 15 dental faculty members (not included in the final study) from two dental colleges in North India participated in the pilot phase in December 2024. Participants were asked to complete the questionnaire and provide feedback via open-ended questions on aspects such as wording ambiguity, relevance of items, and ease of understanding. Cronbach's alpha was calculated to assess internal consistency, yielding an overall value of 0.82 (Section 1:0.79; Section 2:0.81; Section 3:0.84), indicating good reliability for a short scale. Minor revisions were made based on feedback, including rephrasing two items for neutrality (such as clarifying "AI-driven simulations" to avoid technical jargon) and ensuring cultural appropriateness for the Indian context. Content validity was confirmed through expert review by the development team, and face validity was confirmed by pilot participants. No major structural changes were observed.

The target population consisted of full-time and part-time faculty members from dental colleges in North India, specifically from states such as Delhi, Uttar Pradesh, Haryana, Punjab, and Rajasthan. Inclusion criteria were: (1) current employment as a faculty in orthodontics or related interdisciplinary dental departments, (2) at least two years of teaching experience, and (3) willingness to participate voluntarily. The exclusion criteria included non-faculty roles or those without direct involvement in dental education.

A convenience sampling strategy is employed. The sample size was calculated using G*Power software (version 3.1.9.7; Heinrich-Heine-Universität Düsseldorf, Düsseldorf, Germany). The sample size for this survey was estimated a priori using a single-population proportion formula. The calculation was based on the following parameters: 95% confidence level (Zα/2 = 1.96), 5% margin of error (d = 0.05), and expected proportion (p) of awareness of 72% (0.72), derived from a prior study investigating AI awareness among dental professionals [4]. This proportion was selected because it yielded the maximum sample size, ensuring a conservative estimate. The initial calculations indicated a minimum sample size of 235 participants. To account for potential non-response and incomplete submission, the survey was distributed to 410 faculty members from different dental institutions. Contact details were sourced from institutional directories and professional networks, ensuring representation from the government, private, and deemed universities.

The questionnaire was distributed online via WhatsApp, leveraging its widespread use among Indian professionals for efficient and cost-effective dissemination. A Google Forms link was embedded in personalized messages, accompanied by an informed consent statement outlining the study's purpose, voluntary nature, anonymity, and the right to withdraw. Distribution occurred in batches from January 15 to March 15, 2025, with two reminder messages sent at two-week intervals to the non-respondents. Responses were automatically collected through Google Forms, which timestamped submissions and prevented duplicate entries through IP tracking. In total, 410 invitations were sent. Data were downloaded as a comma-separated values (CSV) file and stored securely on a password-protected server that was accessible only to the research team.

Data were compiled in Microsoft Excel (Microsoft, Redmond, WA, USA) and subsequently analyzed using SPSS software (version 26; IBM Corporation, Armonk, NY, USA). Normality of the distribution was verified using the Shapiro-Wilk test. Continuous variables, such as age and years of experience, are presented as mean and standard deviation, while categorical variables, including sex, faculty type, and specialty, are summarized as frequencies and percentages. Outcome measures (opportunity, challenges, strategy) were expressed as mean scores and compared across sex and faculty type using independent t-tests and across interdisciplinary specialties using one-way analysis of variance (ANOVA), with a significance threshold set at p < 0.05. Correlation analyses included Pearson’s correlation for continuous variables and point-biserial correlations for categorical variables.

## Results

Two hundred fifty (61%) dental faculty members responded, with 140 (56%) males and a mean age of 38.11 ± 5.08 years. Male participants had more professional experience (7.46 ± 2.63 years) compared to females (6.91 ± 2.38 years). Orthodontists represented the largest specialty group, followed by prosthodontists, while pedodontists and periodontists constituted equal number. The distribution of faculty types was nearly equal, with clinician-academicians slightly outnumbering academicians (Table 1).

**Table 1:** Basic characteristics of population. Sex, dental specialty and type of dental faculty are presented as frequency (n) and percentage (%), where n denotes number of participants, age and experience are presented as mean ± standard deviation (SD).

Parameters	Category	Value
Sex n (%)	Male	140 (56%)
	Female	110 (44%)
Age in years (Mean ± SD)	Overall	37.62 ± 4.6
	Male	38.11 ± 5.08
	Female	37 ± 3.84
Experience in years (Mean ± SD)	Overall	7.22 ± 2.54
	Male	7.46 ± 2.63
	Female	6.91 ± 2.38
Dental speciality n (%)	Orthodontists	90 (36%)
	Prosthodontists	70 (28%)
	Pedodontists	45 (18%)
	Periodontists	45 (18%)
Dental faculty type n (%)	Clinician-academicians	130 (52%)
	Academicians	120 (48%)

Female faculty members reported significantly higher mean scores for both opportunity (mean = 3.14, p = 0.048) and challenges (mean = 3.41, p = 0.029) compared to their male counterparts (opportunity: mean = 2.82; challenges: mean = 3.14). In contrast, no significant difference was observed in strategy perceptions (p = 0.179), with males reporting a slightly higher mean score (3.32) than females (3.14). These results suggest that sex may influence the perception of opportunities and challenges in AI integration, whereas strategic approaches appear comparable (Table 2).

**Table 2:** Scores for all three outcome measures were compared between females and males using independent t-tests. Scores are presented as mean and standard deviation (SD), *p < 0.05 denotes statistical significance using independent t-test.

Outcome measures	Females		Males		t value	p-value
	Mean	SD	Mean	SD		
Opportunity	3.13	1.14	2.82	1.31	1.98	0.048*
Challenges	3.40	0.78	3.14	1.06	2.20	0.029*
Strategy	3.13	0.97	3.32	1.14	-1.35	0.179

Full-time academicians reported significantly higher challenge scores than faculty with combined academic and clinical roles (clinician-academicians), with this difference being statistically significant (p = 0.013). By contrast, no significant differences were observed between the groups in their perceptions of opportunities (p = 0.601) or strategies (p = 0.654). These results suggest that while both groups share similar views on opportunities and strategies, full-time academicians perceive significantly greater challenges in the measured context than their dual-role colleagues (Table 3).

**Table 3:** Scores for all three outcome measures were compared between academicians and clinician-academicians using independent t-tests. Scores are presented as mean and standard deviation (SD), *p < 0.05 denotes statistical significance using independent t-test.

Outcome measures	Academicians		Clinician-academicians		t value	p-value
	Mean	SD	Mean	SD		
Opportunity	2.91	1.15	3.00	1.33	-0.52	0.601
Challenges	3.41	0.81	3.11	1.05	2.51	0.013*
Strategy	3.20	1.08	3.26	1.06	-0.44	0.654

One-way ANOVA revealed statistically significant differences among interdisciplinary specialties in perceptions of opportunities (p = 0.001) and challenges (p = 0.004). Orthodontists demonstrated the highest mean opportunity scores (mean = 3.33), whereas pedodontists reported the lowest (mean = 2.56). For challenges, prosthodontists showed the highest levels (mean = 3.57), whereas orthodontists reported the lowest levels (mean = 3.06). By contrast, no significant differences emerged in strategy perceptions across specialties (p = 0.397). These findings suggest that professional specialization significantly influences the perception of opportunities and challenges in dental practice, while strategic approaches remain consistent across different disciplines (Table 4).

**Table 4:** Scores for all three outcome measures were compared between dental specialties using one-way analysis of variance (ANOVA) test. Scores are presented as mean and standard deviation (SD), *p < 0.05 denotes statistical significance using one-way ANOVA test.

Outcome measures	Orthodontists		Pedodontists		Periodontists		Prosthodontists		F value	p-value
	Mean	SD	Mean	SD	Mean	SD	Mean	SD		
Opportunity	3.33	0.82	2.55	1.51	3.00	0.95	2.71	1.54	5.43	0.001*
Challenges	3.05	0.97	3.33	1.06	3.11	1.00	3.57	0.73	4.47	0.004*
Strategy	3.16	1.17	3.44	0.69	3.33	0.67	3.14	1.31	0.99	0.397

The anticipated correlations revealed distinct patterns based on demographic variables. Weak positive correlations are expected between both experience and age and perceptions of opportunity and strategy, suggesting that senior faculty, possibly due to greater exposure to technological evolution, may recognize more potential and possess clearer implementation approaches. By contrast, a very weak negative correlation with challenges implies that they may feel less impeded. Notably, gender showed negligible and non-significant correlations across all parameters, indicating that perceptions were independent of sex. The strongest findings emerged with professional designation: Academicians are anticipated to demonstrate a weak-to-moderate negative correlation with opportunity and strategy, reflecting their potentially greater engagement with innovation, while clinician-academicians may report a weak positive correlation with challenges, likely due to practical clinical constraints and integration hurdles. These trends highlight the influential role of professional roles and seniority in shaping faculties’ perspectives (Table 5).

**Table 5:** Correlation analysis between outcome measures and independent variables. *p < 0.05 denotes statistical significance using Pearson correlation for experience and age and point-biserial correlation for sex and faculty type. r value: 0.00 – 0.19 suggests very weak correlation, r value: 0.20 – 0.39 suggests weak correlation, positive (+) values indicate a direct relationship, whereas negative (–) values indicate an inverse relationship.

Measures	Test statistics	Experience	Age	Sex	Faculty type
Opportunity	r value	0.25	0.18	0.08	-0.32
	p-value	0.001*	0.006*	0.215	0.001*
Challenges	r value	-0.15	-0.10	-0.05	0.22
	p-value	0.018*	0.112	0.438	0.001*
Strategy	r value	0.30	0.22	0.12	-0.28
	p-value	0.001*	0.001*	0.063	0.001*

## Discussion

The findings of this study provided valuable insights into faculty perspectives on AI integration in orthodontic and interdisciplinary dental curricula, highlighting opportunities, challenges, and strategies, while revealing variations influenced by demographic and professional factors. Overall, the high mean scores across sections indicated a generally positive outlook on AI's potential to enhance dental education, aligning with the growing recognition of AI as a transformative tool in dentistry [2,3]. For instance, AI-driven diagnostic tools and personalized learning systems have been perceived as key opportunities, consistent with broader trends in which AI improves diagnostic accuracy and educational outcomes [2].

Sex differences emerged prominently, with female faculty reporting significantly higher perceptions of both opportunities and challenges in AI integration. This suggests that females may be more attuned to the dual-edged nature of AI, embracing its innovative potential while being acutely aware of barriers, such as ethical concerns and training gaps. Similar findings were reported by Ghasemian et al. [7], who found that female participants favored AI inclusion in dental education. Previous research supports this pattern; a study on dental students' attitudes found that female participants favored AI inclusion in education more than males, potentially due to a greater emphasis on patient-centered and educational applications [7,8]. However, contrasting evidence indicates that sex disparities sometimes favor males in AI readiness, highlighting the need for context-specific interpretations, such as in Indian dental settings, where cultural factors might influence perceptions [9]. These findings underscore the importance of sex-sensitive approaches to AI adoption to harness diverse viewpoints.

Regarding faculty type, full-time academicians perceived greater challenges than those with combined academic-clinical roles, while views on opportunities and strategies remained similar. This disparity may stem from academicians' deeper involvement in curriculum design, making them more cognizant of barriers, such as limited AI training and infrastructural deficits in educational settings. In contrast, clinician-academicians exposed to practical AI applications in clinics might view challenges as more surmountable through real-world integration. Supporting studies on AI in medical and dental education highlight that pure academics often report higher barriers due to a lack of hands-on experience, whereas hybrid roles foster greater acceptance by bridging theory and practice [10,11]. Al-Zubaidi et al. [11] conducted a survey to explore the preparedness of faculty members to incorporate AI in dental education, concluding that most faculty members wanted AI to be integrated into dental education. However, qualitative exploration of faculty perceptions emphasized training gaps among educators, reinforcing the need for targeted development programs to align academic and clinical perspectives.

Specialty-based variations were significant for opportunities and challenges but not for strategies. Orthodontists exhibited the highest opportunity scores and lowest challenge perceptions, likely because of the heavy reliance of orthodontics on imaging and predictive modeling, where AI excels in tools such as cephalometric analysis and treatment simulation [2,12]. Conversely, prosthodontists reported the greatest challenges, possibly attributable to the field's emphasis on hands-on fabrication and customization, where AI integration faces hurdles in precision and material science [13]. Pedodontists scored the lowest on opportunities, reflecting pediatric dentistry's focus on behavioral management, where AI's role remains nascent [14]. These differences align with prior research, which found orthodontics to have the highest AI utilization (80%), improving diagnosis and efficiency, while other specialties such as prosthodontics express concerns over technical limitations [15]. Another study on AI perceptions among dental professionals noted specialty-specific barriers, supporting tailored curriculum redesigns to address these disparities [16].

Correlation analyses revealed weak positive correlations between age/experience and perceptions of opportunities and strategies, along with a weak negative correlation with challenges, indicating that senior faculty members are more receptive to AI. This may result from the accumulated exposure to technological advancements, enabling faculty members to envision AI's benefits while downplaying obstacles. In contrast, younger faculty members might overestimate challenges due to their limited practical experience. Abdullah et al. [10] reported that age groups of 36-45 years demonstrated more positive attitudes towards AI adoption. These patterns are corroborated by a previous study in which greater professional tenure correlates with higher AI acceptance [17]. A review on AI acceptance among healthcare professionals similarly found that experience fosters positive attitudes, reducing perceived barriers, such as overreliance on technology [18].

Clinical implications

These results have practical implications for clinical dental practice. By integrating AI into curricula, future practitioners, especially orthodontics practitioners, can leverage tools for enhanced diagnostic precision, leading to improved treatment outcomes and reduced errors in patient care. Addressing challenges through faculty training could minimize ethical risks, such as data privacy breaches, ensuring AI support, rather than replacing clinical judgment. Interdisciplinary strategies may foster collaboration, enabling AI-driven personalized treatment across specialties, ultimately elevating patient satisfaction and efficiency in technology-driven practices.

Future recommendations

Based on these results, future efforts should prioritize the development of specialized AI training modules tailored to dental specialties, particularly emphasizing orthodontics for rapid adoption and prosthodontics/pedodontics for overcoming barriers. Institutions are recommended to implement pilot programs for AI tools in curricula, incorporate interdisciplinary workshops to foster collaboration, and address ethical concerns such as data privacy. Longitudinal studies tracking AI integration outcomes over time would provide deeper insights, while policy recommendations include allocating resources for infrastructure upgrades and faculty development in underserved regions, such as North India. Additionally, encouraging partnerships with AI experts could accelerate personalized learning systems, ensure equitable access, and prepare students for evolving dental practices.

Limitations

This study has several limitations. Convenience sampling from North India may limit the generalizability to other regions or countries with varying infrastructural and cultural contexts. Reliance on self-reported data and no triangulation (e.g., interviews, focus groups) introduces a potential response bias, and the cross-sectional design precludes causal inferences. The numerical scale, while simple, may not capture nuanced opinions. Future research should incorporate longitudinal and qualitative methods to gain deeper insight.

## Conclusions

This study revealed faculty perspectives on AI integration in orthodontic and interdisciplinary dental curricula, emphasizing opportunities for AI-driven diagnostics and personalized learning, alongside challenges such as training deficiencies and ethical concerns. Female faculty members reported higher awareness of both opportunities and barriers, while orthodontists were most optimistic and prosthodontists noted significant challenges. Full-time academicians perceived greater obstacles than clinician-academicians did. Specialty and experience shaped perceptions, with senior faculty members showing greater AI receptivity. These findings highlight the need for targeted curriculum enhancements and faculty training to facilitate AI adoption and prepare students for technology-driven dental practice despite limitations such as regional sampling and self-reported data.

## Figures and Tables

**Figure 1:**
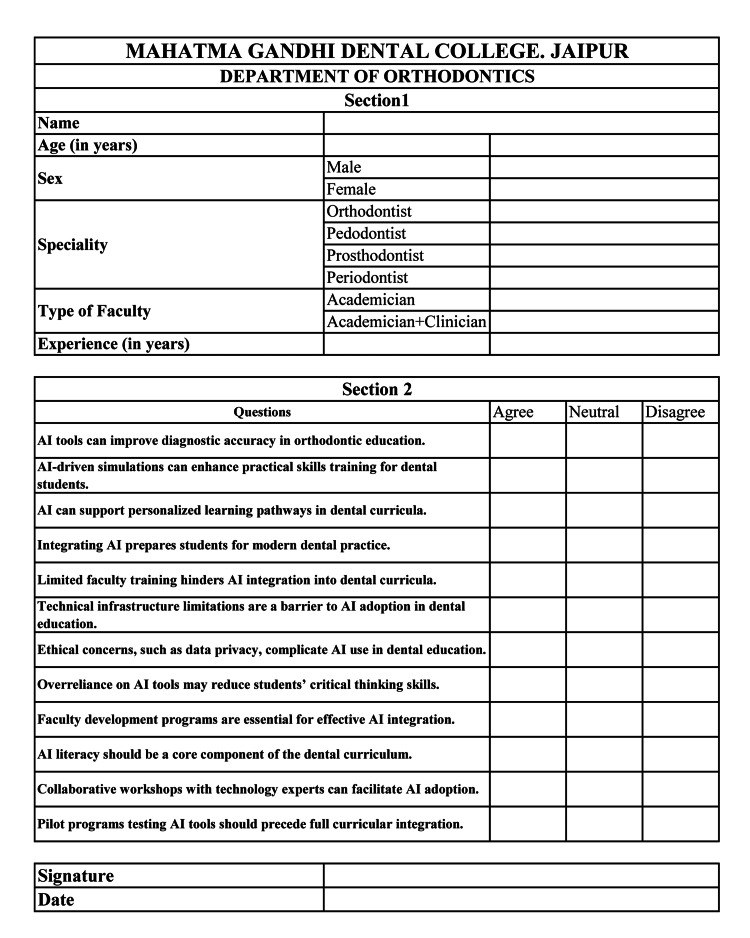
The original study questionnaire was developed and utilized for data collection in this study. Credit: Priyanka Agarwal, Rupali Rupali, Angel Angel, Chand Sawhney, Joyshree Chutia.
